# Hemorrhagic stroke after Epley maneuver: a case report

**DOI:** 10.1186/s40463-018-0268-9

**Published:** 2018-04-10

**Authors:** Paige Moore, Trung Le, Brian Blakley, Jason Beiko, Eric Meen

**Affiliations:** 1 Department of Otolaryngology - Head & Neck Surgery, University of Manitoba, GB421 - 820 Sherbrook Street, Winnipeg, MB R3A 1R9 Canada; 2 Department of Otolaryngology - Head & Neck Surgery, Sunnybrook Health Sciences Centre, Toronto, ON Canada; 3 Department of Neurosurgery, University of Manitoba, Winnipeg, MB Canada

**Keywords:** Benign paroxysmal positional vertigo, BPPV, Stroke, Hemorrhage, Adverse outcome, Risk, Epley maneuver, Valsalva maneuver, Nausea, Vomiting, Intraparenchymal hemorrhage

## Abstract

**Background:**

This is the first case to our knowledge of a serious adverse event following the Epley maneuver, which is the treatment of choice for benign paroxysmal positional vertigo (BPPV), the most common vestibular disorder in adults.

**Case presentation:**

A 77 year old female presented for outpatient evaluation of vertigo at a tertiary otolaryngology clinic. She was found to have BPPV clinically, and elected to have a particle repositioning maneuver (Epley maneuver) performed in clinic. Immediately following Epley maneuver, she had severe nausea and vomiting, with evolving visual changes. A CT angiogram of the brain was performed urgently through the emergency department and demonstrated an acute intraparenchymal hemorrhage in the occipital lobe. After medical stabilization and rehabilitation, the patient continues to have a permanent visual field deficit.

**Conclusion:**

The Epley maneuver is safe and effective, and there are no prior reports of serious adverse events associated with its use. This case, in which a patient experienced a hemorrhagic stroke after undergoing the Epley maneuver, is the first and sole case in the medical literature of an Epley-associated serious adverse event. The indirect causation and extreme rarity of this event do not warrant any change to patterns of practice.

## Background

Benign paroxysmal positional vertigo (BPPV) is characterized by brief episodes of vertigo associated with changes in head position [1], and is the most common vestibular disorder [2]. Although occasionally from secondary or acquired etiologies (as in post-trauma, vestibular neuritis, labyrinthitis, vertebrobasilar ischemia), the majority of cases are idiopathic in nature.

BPPV diagnosis is typically confirmed by a Dix-Hallpike maneuver. This diagnosis is probable when the patients’ symptoms are reproduced with moving from sitting to a supine position, with the head in hanging position and turned 45° to the affected side. The diagnosis becomes definite when the Dix-Hallpike test elicits a fatiguable, reversible, torsional geotropic, paroxysmal nystagmus, accompanied by vertigo [3].

Treatment of BPPV is primarily based on particle repositioning, by directing the canaliths into the utricle where they no longer stimulate the vestibular system. This can be achieved through various canalith repositioning maneuvers, of which the most popular is the Epley maneuver.

Although certain forms of neck manipulation are felt to exacerbate ischemic strokes by cervical artery dissection [3], the Epley maneuver itself has strong evidence for safety and efficacy, with no serious adverse effects reported in the literature [1, 4, 5].

## Case presentation

A 77-year-old woman presented for otolaryngology consultation. She complained of approximately 3 months of positional spinning sensation, worse with lying flat, or on her left side. The episodes lasted several seconds before resolving spontaneously. She had similar problems approximately 15 months prior, which had resolved completely and spontaneously after three weeks. Her current state left her feeling unable to lie flat and she had cancelled cataract surgery as a result. She had no new vision complaints.

Her relevant past medical history included a remote diagnosis of hypertension. On presentation to our service she had been normotensive without medications. Additionally, she had a right-sided cataract with stable vision. There was no previous history of stroke or ischemic heart disease. She took no medications, and had no allergies. She had no history of smoking or alcohol use. She lived independently and was very active.

On exam, there was no spontaneous or gaze-evoked nystagmus. The vestibuloocular reflex was intact, and there was no post head-shake nystagmus. Dix-Hallpike testing was strongly positive to the left, with subjective vertigo and fatigable nystagmus. Given the patient’s good functional status and overall health, she was felt to be an excellent candidate for the Epley maneuver, which was offered and agreed to, with slight subjective dizziness at all stations. Upon completion of the Epley maneuver, she experienced severe nausea and forceful vomiting for several minutes. She then complained of blurred vision and continued retching for an additional 5–10 min. Vital signs obtained in clinic were a blood pressure of 178/58, and pulse of 66. The symptoms of vertigo, severe nausea, and blurred vision persisted after 30 min and the patient was transferred to the emergency department for emergent assessment, including CT angiogram of the brain.

**Fig. 1:**
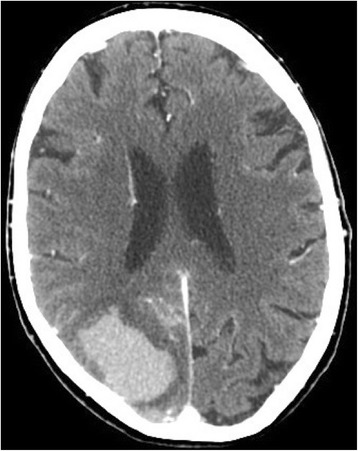
Axial CT angiogram demonstrating acute hemorrhage to the right occipitoparietal region, with surrounding edema

Contrast-enhanced CT angiography of the brain was performed (Fig. 1). This demonstrated a 4.3 × 2.5 cm acute intraparenchymal hemorrhage in the right occipital lobe, and a small amount of subarachnoid hemorrhage in the right basal cistern and cerebellopontine angle. There was mild mass effect and surrounding edema, but no significant midline shift. Atherosclerosis to the common carotid bifurcations was present bilaterally, without significant stenosis. The vertebral arteries and circle of Willis were widely patent without dissection, thrombosis, or aneurysm.

A neurosurgeon reviewed the images and felt there was no role for surgery based on the stability of the patient’s neurological exam. Internal medicine was consulted and the patient was admitted to their service for monitoring of clinical status and management of hypertension. At the time of admission, the only pertinent neurologic finding was a left homonymous hemianopsia. The remainder of the neurologic exam was normal. Blood pressure was 187/82 at Emergency triage, but returned to the normal range shortly after admission where it remained, without antihypertensive therapy, for the duration of her stay in hospital.

Her admission was uncomplicated, with subjective improvements to her vision, and was she transferred to a stroke rehabilitation program 13 days after the initial event.

**Fig. 2:**
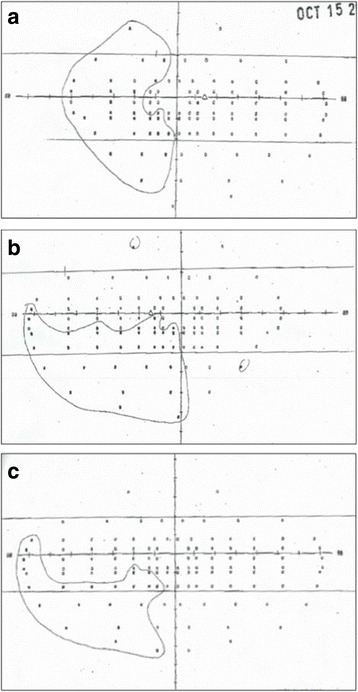
Visual field testing results 16 months after initial event, demonstrating areas of deficit (encircled) in the **a**) right eye; **b**) left eye; **c**) binocular vision

Stroke rehabilitation physicians noted an ongoing left hemianopsia, and mild left-sided neglect 2 weeks after the stroke, but the patient was safe and independent with her activities of daily living. She was discharged home 27 days after her stroke. Ophthalmologic testing 15 months later revealed a persistent binocular deficit to the left inferior visual field, and 20/30 binocular acuity (Fig. 2).

## Discussion

There is ongoing debate about the relationship between neck manipulation and cervical artery dissection, which can lead to ischemic strokes. The vast majority of this literature is chiropractic or physiatric in nature, and pertains to brisk and jarring cervical manipulation, unlike particle repositioning maneuvers. Physiologically, mechanical forces can lead to intimal injuries of the internal carotid and vertebral arteries, as evidenced by known stroke triggers such as neck rotation and extension, implying an inherent fragility of the intimal wall [4]. However, it is unclear whether there may be preexisting vascular disease in the affected population [5] . Although some case-control studies have suggested an association between cervical manipulation therapy and cervical artery dissection (typically vertebral artery dissection as opposed to internal carotid artery dissection), there is insufficient evidence to establish a causal relationship between the two^,^ [6–8]. Due to the very low incidence of both internal carotid artery and vertebral artery dissection (2.5 to 3 per 100,000 patients and 1 to 1.5 per 100,000 patients, respectively) [6] and the limitations of the aforementioned case-control literature, the incidence of cervical manipulation therapy-associated cervical artery dissection has not been established.

The Epley maneuver is very efficacious, with many patients converting to a negative Dix-Hallpike and experiencing a complete resolution of vertigo after the initial treatment [1, 2, 9–11]. The natural history of BPPV tends towards spontaneous resolution without treatment in up to 84% of cases [12]. In addition, the only reported adverse events associated with the Epley maneuver are nausea and vomiting, with rates of 16.7–32% [1, 10, 11]. There are no reports in the literature of ischemic or hemorrhagic stroke associated with particle repositioning maneuvers.

We have presented the first case to our knowledge of intraparenchymal hemorrhage related to the Epley maneuver, resulting in a permanent visual field deficit. Causation is uncertain in this case. It is possible that the patient’s forceful retching and vomiting caused a significant increase in intracranial pressure by way of repeated Valsalva Maneuvers [13], which then resulted in rupture of a small intracerebral artery [14]. Supporting this is that the occipital lobe (the location of our patient’s hemorrhage) is a common location for intracerebral hemorrhage associated with acute hypertensive changes [15].

Another possible mechanism is that the patient’s intense nausea and vomiting were elicited by a stroke itself occurring during particle repositioning – it is well established that nausea and vomiting are common symptoms at the onset of hemorrhagic stroke [16]. It is clear that a history of long-standing hypertension (which, despite having reportedly being controlled off of medications, was previously present in our patient) is a risk factor for intracerebral hemorrhage due to gradual vessel degeneration over time predisposing to vessel rupture [14]. There was no evidence of cervical artery dissection, thrombosis, or ischemia, in our patient, which is the typical pathophysiology of neck manipulation-induced stroke [6, 17].

## Conclusion

The Epley maneuver is commonly performed, and is safe and effective in otolaryngology practice. This is the sole case present in the medical literature of an Epley maneuver-associated serious adverse event, and specific causation in our patient’s case is unclear. Regardless of our patient’s case, careful selection of appropriate patients remains important when deciding on whom to perform the Epley maneuver, particularly in elderly patients with comorbidities. The indirect causation and extreme rarity of this event do not warrant change to practitioner patterns of practice.

## Abbreviations

BPPV: benign paroxysmal positional vertigo
CT: computed tomography
ENT: Ear, nose and throat surgery (aka otolaryngology- head & neck surgery)
